# Shaping the Design Features of a Dynamometer for Measuring Resistance Biaxial Components of Symmetrical Coulters

**DOI:** 10.3390/s22010272

**Published:** 2021-12-30

**Authors:** Jacek Marcinkiewicz, Mikołaj Spadło, Zaneta Staszak, Jarosław Selech

**Affiliations:** 1 Faculty of Civil and Transport Engineering, Poznan University of Technology, 60-965 Poznan, Poland; jacek.marcinkiewicz@put.poznan.pl (J.M.); zaneta.staszak@put.poznan.pl (Z.S.); 2 Department of Mechanical Engineering, Institute of Machine Design, Poznan University of Technology, 60-965 Poznan, Poland; mikolaj.spadlo@put.poznan.pl

**Keywords:** FEM, force transducer, soil, calibration, measuring sensitivity, strain gauge

## Abstract

The article lays out the methodology for shaping the design features of a strain gauge transducer, which would make it possible to study forces and torques generated during the operation of symmetrical seeder coulters. The transducers that have been known up until now cannot be used to determine forces and torques for the coulter configuration adopted by the authors. For this purpose, the design of the transducer in the form of strain gauge beams was used to ensure the accumulated stress concentration. A detailed design was presented in the form of a 3D model, along with a transducer body manufactured on its basis, including the method for arranging the strain gauges thereon. Moreover, the article discusses the methodology of processing voltage signals obtained from component loads. Particular attention was paid to the methodology of determining the load capacity of the transducer structure, based on finite element method (FEM). This made it possible to choose a transducer geometry providing the expected measurement sensitivity and, at the same time, maintaining the best linearity of indications, insignificant coupling error, and a broad measurement range. The article also presents the characteristics of the transducer calibration process and a description of a special test stand designed for this purpose. The transducer developed within the scope of this work provides very high precision of load spectrum reads, thus enabling the performance of a detailed fatigue analysis of the tested designs. Additionally, the versatility it offers makes it easy to adapt to many existing test stands, which is a significant advantage because it eliminates the need to build new test stands.

## 1. Introduction

Contemporary agricultural machines must ensure high quality of the performed work demonstrated in the performance and precision in the cultivation of soil. Therefore, laboratory studies making it possible to better understand and identify the phenomena occurring when they operate in the soil are crucial [1,2,3,4]. To verify the design assumptions, experimental testing related to their fatigue life is conducted. These tests can be divided into three basic types: field tests, track tests, and bench tests [5].

Bench tests were carried out on properly designed test stands, e.g., soil channels [6,7,8,9,10,11] or rotating bowls [12,13] which made it possible to measure the parameters related to the operation of a tool, i.e., force, torques, and wear. Depending on the type (i.e., shape, size, fixing method) of the tested element, the structure and dimensions of test stands differ. However, they all enable experiments to be conducted for small tools which make up a larger structure [14,15,16,17,18,19,20]. Such structural diversity of test stands makes it possible to measure characteristic parameters for the given agricultural machine at the selected nodes [21,22,23,24,25].

**Figure 1:**
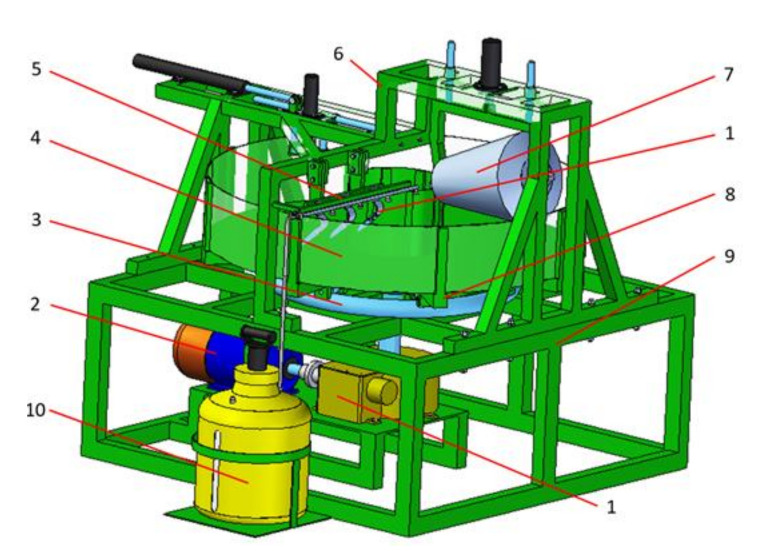
Test stand: 1—transmission, 2—motor, 3—running rail, 4—bowl, 5—sample holder, 6—supporting frame, 7—compacting roller, 8—bowl frame, 9—main frame, 10—wetting system [12].

A well-designed and prepared test stand with adequate dimensions and testing capabilities, i.e., automatic control of the tested tool travel or the possibility to measure forces and torques, provides multiple testing capabilities [26,27]. Apart from observation of the way in which the tested tools act on the processed soil, data on forces and torques occurring while they operate can be obtained [28,29]. The proper determination of the forces and torques acting on the tool operating in the soil is very important, both in the design and operational context. It makes it possible to choose the right tractor power and determine fuel consumption [30,31,32,33]. On the other hand, in the design aspect, it prevents the structure from being oversized by supporting decisions regarding the selection of the construction materials depending on the occurring variable strains or deformations [34,35]. Therefore, the construction of a new test stand (Figure 1), developed in the Institute of Machine Design at the Poznan University of Technology, included a number of innovative design solutions. The test stand enables the use of advanced measurement systems, including complex measuring tracks, which make it possible to track the patterns of changing loads in the tested tools. The basic element of such solutions can be a transducer for measuring component loads acting on the tools operating in the ground.

The number of literature references discussing methods of recording loads acting on components operating in the soil, especially on coulters, is relatively small [26]. This issue is far from trivial because coulters are loaded with six components acting simultaneously and three of them play a key role as far as fatigue processes are concerned (longitudinal force, bending moment, vertical force). It is necessary to implement more complex technological solutions in order to enable the measurement of these loads.

Some articles present a method for measuring the six load components based on a system installed between a three-point linkage and the agricultural machine [36,37,38,39,40]. A system of this type cannot be used in the load measurement systems in coulters. This is due to the fact that coulters are characterised by much lower stiffness than plough tools; thus, the scope of the applied forces is considerably smaller. There is a significant risk that the measurements would be subject to the noise caused by natural vibrations of the three-point linkage of the tractor and the load-bearing frame of the seeder or planter to which the coulters would be mounted [41]. In their previous work, the authors of this article presented a test stand and a measurement transducer for large elements of machines operating in the soil, among others for ploughshares, where considerably higher forces are present [26]. The coulters of a sowing unit or cultivator are tools which operate in the soil similarly to a ploughshare. The difference between these tools is due to their size and depth of operation in the soil. Therefore, the use of the previously developed transducers does not allow to obtain reliable information because they are intended for measuring considerably higher forces generated by large working elements of machines. It is necessary to measure forces near the areas in which they appear, as demonstrated in this article.

In order to measure the biaxial components of resistance of the symmetrical coulter assembly, a transducer was developed, manufactured and tested, and then fitted with sensors suited to the size of the tools and the forces they generate. The developed transducer makes it possible to obtain reliable and true results of the measurement of forces and torques occurring on much smaller elements, such as cultivator or seeder coulters. Its versatility makes it fit suitable for use in several existing test stands [1,7,14,42,43], which is a substantial advantage because there is no need to design and build a new test stand. The test was carried out on a rotating bowl test stand.

## 2. The Purpose of the Article

The purpose of the article was to develop a transducer enabling identification of the two main components of excitation of force and torques occurring during the operation of a symmetrical coulter assembly.

In such coulters, the side components can cancel each other out or do not occur at all. This contributes to the elimination of side resistance and thus creates a biaxial arrangement that comprises horizontal and vertical resistance [44,45,46].

**Figure 2:**
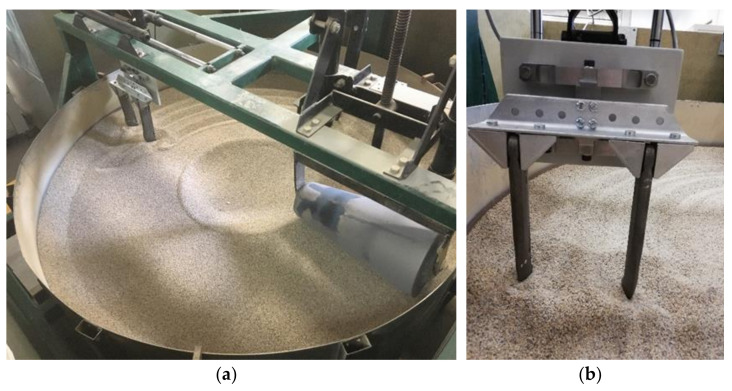
Test stand: (**a**) view of transducer attachment to the tool holder, (**b**) view of symmetrical coulters mount.

The spatial measurement method of dynamic loads with the use of strain gauge measurements was adopted as the basis for the developed transducer design. This made it possible to ensure a compact design installed between the tested tool and the positioning system (Figure 2). The calibration of the transducer was done on a test stand designed especially for the purpose of testing, making it possible to simplify the construction and the operating procedures of the developed measuring device.

## 3. Shaping the Design Features of the Transducer

**Figure 3:**
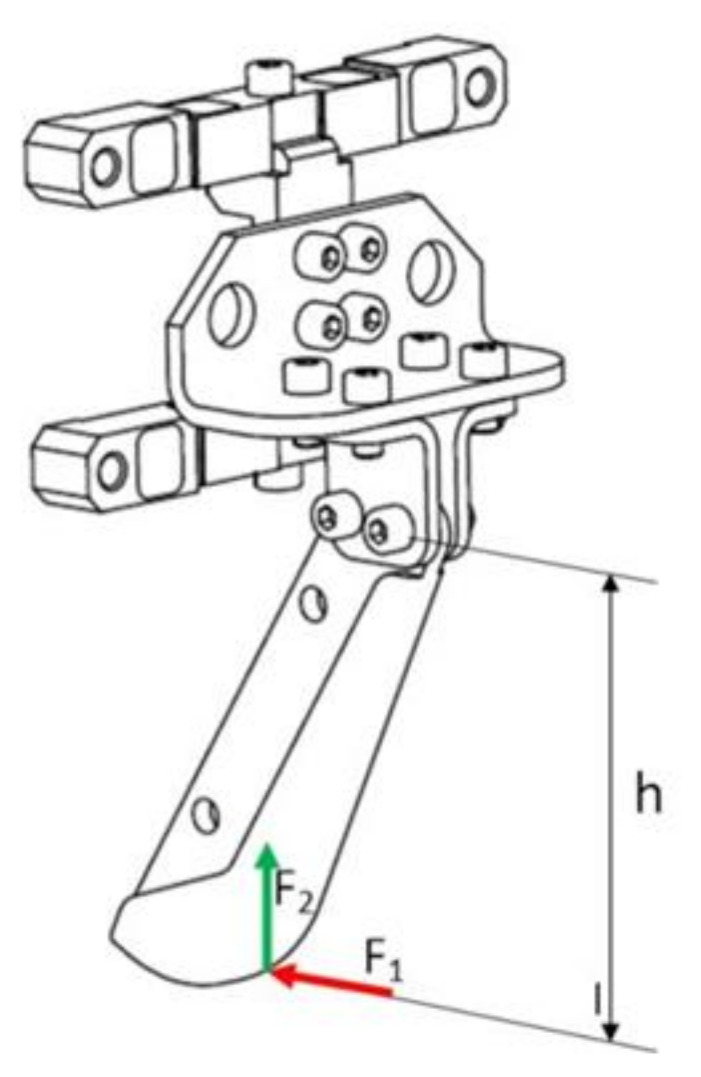
System of loads acting on the coulter.

The basis for the development of the transducer is a system of actual loads acting on a working element in the form of two symmetrically positioned coulters. A coulter is a mechanical system in the form of a flexible beam, which is subjected to loads listed in the figure below (Figure 3). Longitudinal force F_1 generates a bending moment on the “r” arm, which is the main source of strain. Apart from the longitudinal force, there is also vertical force F_2. In order to record these loads, it is necessary to develop a dedicated measuring system, i.e., one that

(1). can measure forces within a specific range (from 10 to 600 N),
(2). has a natural frequency of vibration that is higher than usable frequencies (for resonance prevention).

The proper selection of geometrical features of the transducer structure is a complex process, which requires multiple criteria to be taken into account. These include the need to ensure adequate measurement precision with the best possible linearity of indications and insignificant coupling error to ensure the highest measurement range. In the light of the above, it could be concluded that solutions which guarantee the highest possible sensitivity while ensuring the highest stiffness of the system are sought for [25]. In fact, however, these are two mutually exclusive requirements which cannot be satisfied at the same time. In such cases, it is necessary to select the right design features for properly determined measurement ranges, so that the most important values of the measured parameters are within the threshold.

Based on the previous experimental tests and literature, it was found that resistance during operation ranges from 5 N to 500 N [26]. It was therefore determined that, in order to allow full and correct identification, a dynamic parameter meter of low values should be used, for which the following preliminary assumptions were made:

(1). force along the Z axis − F_z = 450 N,
(2). force along the Y axis − F_y = 200 N,
(3). torque about the X axis − M_x = 200 Nm.

**Figure 4:**
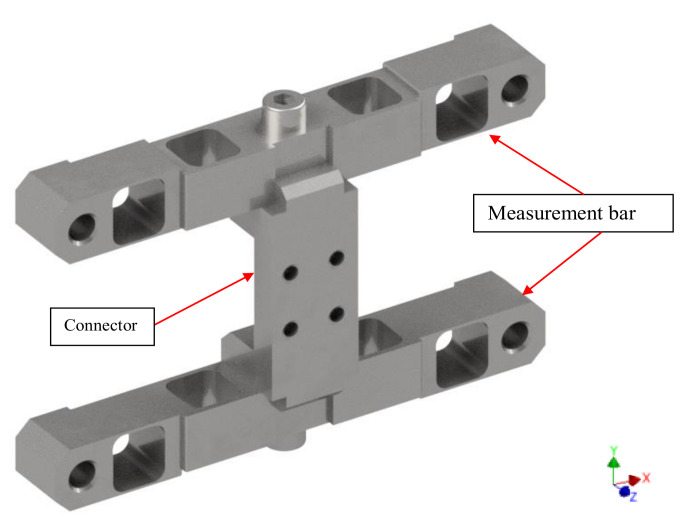
Overview of the transducer.

**Figure 5:**
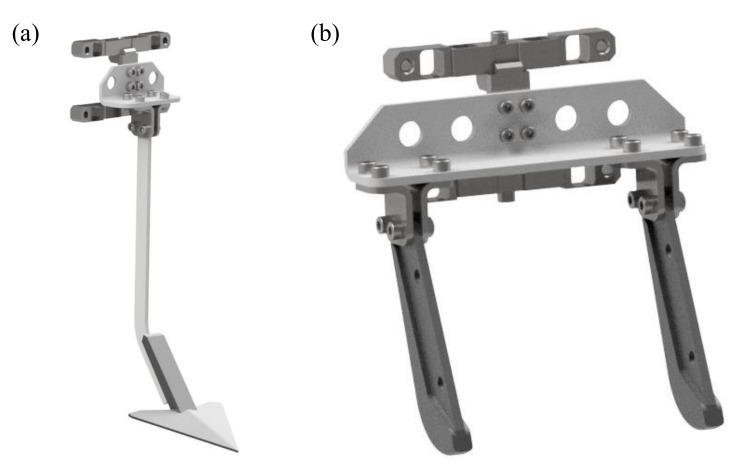
Transducer in configuration with various tool types; (**a**) coulter terminated with a goosefoot, (**b**) two coulters of a seeder.

For the purpose of measurement of such small force components, it was decided that the spatial measurement method of dynamic loads with the use of crossbars should be implemented. In the adopted solution, a system comprising three elements: two measurement bars and a connector arranged as in Figure 4 was proposed. The system elements were designed so that they can be reconfigured depending on the type of agricultural tool (Figure 5). At the same time, this solution made it possible to simplify the production process of precision measurement bars and thus decreased their cost of production.

In terms of the design, the top and bottom bars are carbon copies of each other. They were designed as cubic elements with a square cross-section and dimensions of 20 mm × 20 mm with four drilled out rectangular holes. The drilled-out holes were positioned symmetrically in pairs in an arrangement alternately rotated by 90°. Moreover, the construction of the bars was supplemented with externally positioned installation holes, which constitute the installation area of the transducer to the body of the drive unit of the test stand (Figure 2). Stiffness adjustment of the designed force meter results from the change of dimensions of the rectangular holes. Such an approach makes it possible to develop an entire series of transducers, where a change of a single geometric parameter (wall thickness) makes it possible to properly tune the transducer in accordance with the planned measurements.

The unique alignment of measurement bars in relation to one another was achieved thanks to the stiff structure of the thick-walled connector. The connector was developed as the main installation element for the tested components and for this purpose, in its basic version, four M10 installation holes were placed and arranged on a square layout with a 30 mm side length. Furthermore, in its structure, at the point of contact with the bars, special shape connections were introduced. They ensure the required joint stiffness for the built structure with the use of just a single bolt node. The adopted solutions enable easy and quick disassembly and replacement, making the sensor a universal solution that can be used with many types of agricultural tools. Prepared in this way, the structure enables easy installation and removal of the tested tools.

17-4PH steel was used as the construction material, which has the elastic modulus E = 1.96 × 1011 N·m^−2^, the Poisson ratio ν = 0.30, the density ρ = 7.85 × 103 kg·m^−3^, and the yield strength σs = 1100 × 106 N·m^−2^.

## 4. Strain Analysis

**Figure 6:**
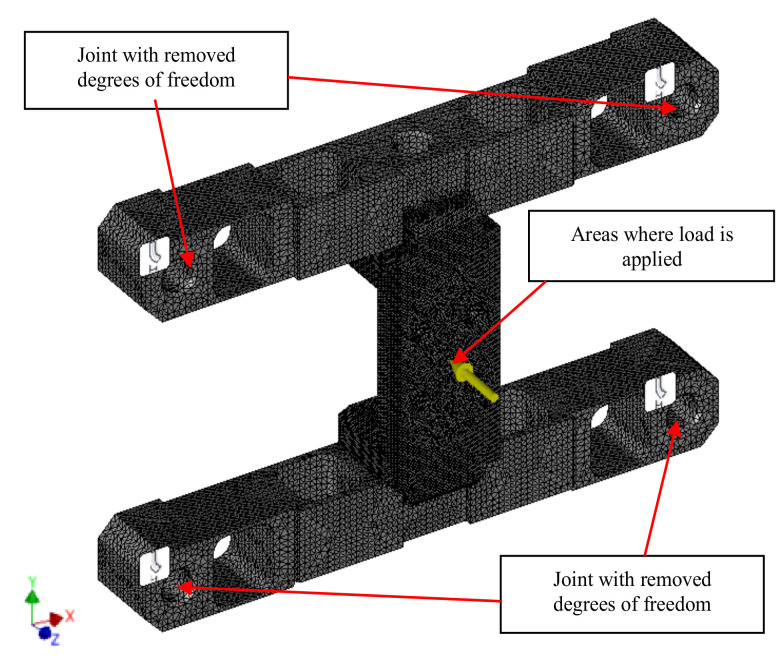
Transducer calculation model.

In order to perform simulation tests, a properly parametrised virtual models were prepared, as presented in Figure 6. Specialist CAD-3D software was used to prepare them. With the use of pre- and postprocessors of graphic engineering interpretation, the calculation model was described with solid elements enabling approximation of operating characteristics of an object in real conditions [47,48]. Based on the adopted transducer construction and the occurring loads, tetrahedral parabolic second-order elements were used in the prepared model. This ensured a more accurate mathematical representation than in the case of linear elements [49]. The degrees of freedom were defined based on the actual operation of the transducer by depriving the nodes around the installation holes of the capability to move along the longitudinal and transverse axes of the transducer. As a result, its contact with the area of tool installation to the test stand was reflected [9]. The capability of the nodes in the installation holes to move along all axes was removed. During the tests, forces and torques were applied to the nodes constituting the face surface of the connector.

As a result of the performed simulation tests (FEM), strain distribution for three cases was obtained, corresponding to the permissible forces and torques. Strain values reduced for the analysed structure were obtained using the von Mises yield criterion. In each simulation experiment, the loads were applied to the surface constituting the agricultural tool installation area.

**Figure 7:**
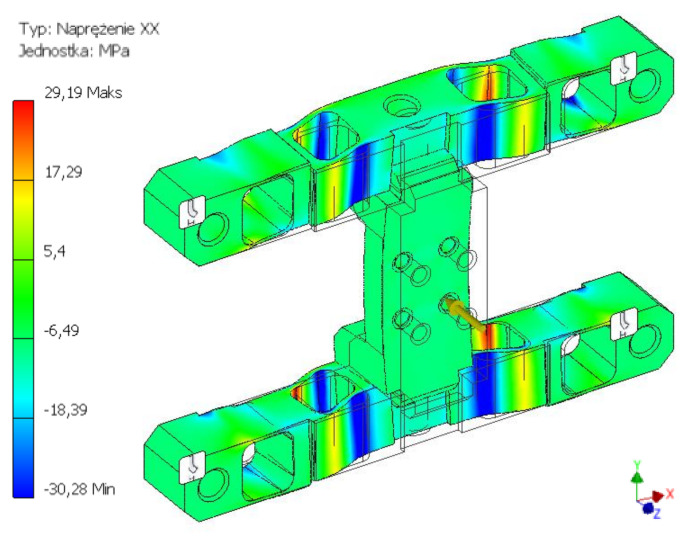
H-M-H strain map for the force value of 450 N acting along the Z axis.

**Figure 8:**
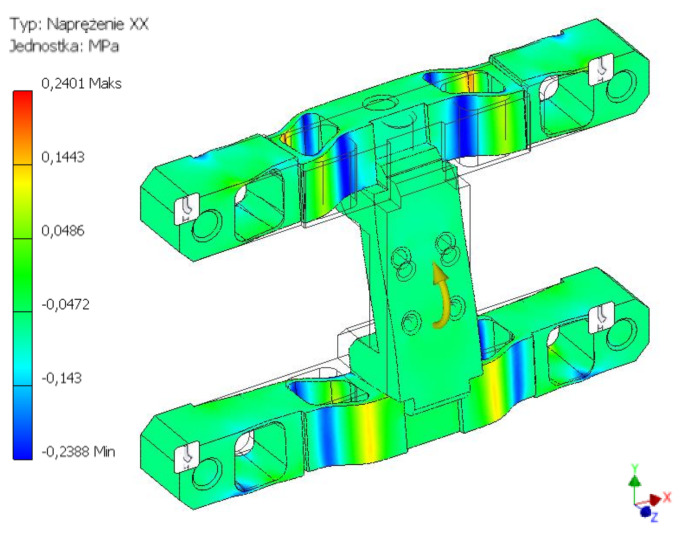
H-M-H strain map for the force value of 150 N acting along the X axis.

**Figure 9:**
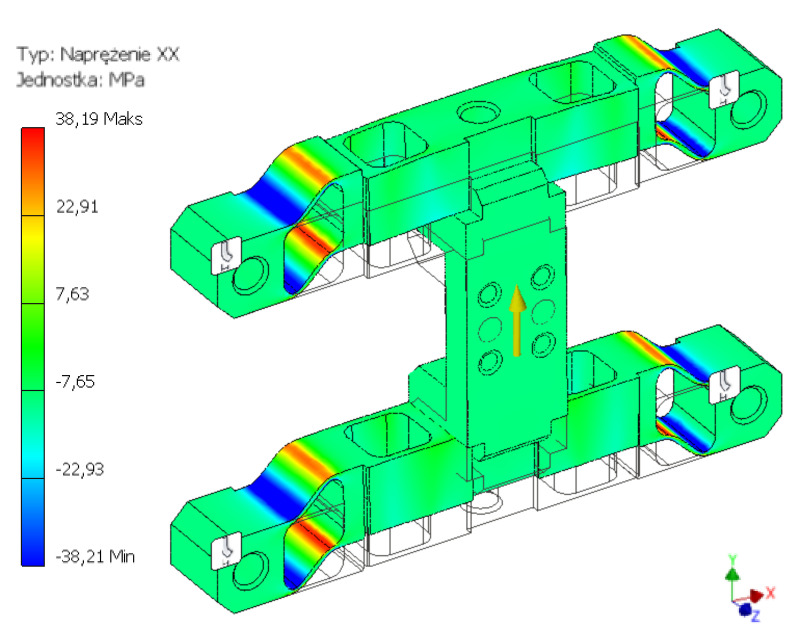
H-M-H strain map for the force value of 200 N acting along the Y axis.

The first of the considered load states concerned the maximum permissible strain of the construction along the Z axis, i.e., perpendicular to the surface. For the adopted force Fz = 450 N, the maximum strain values of 29 MPa were obtained and were concentrated around the internal through holes. The second considered load component was the excitation along the Y axis, i.e., in the direction parallel to the installation surface. The applied load with the value of Fy = 200 N caused a concentration of strain around the outermost holes. The read values did not exceed 39 MPa. The last of the tested states concerned the effects related to the torque moment about the X axis. For the adopted load of Mx = 150 Nm, strains with a maximum value of 0.24 MPa were obtained. The strains were concentrated in the terminal points of the crosswise edge of the connection with the brackets. The results of the analyses of the body structure, presenting the strain distribution caused by the effect of loads in the form of force acting along the Y axis and the torque about the X axis, were provided in figures from Figure 7, Figure 8 and Figure 9.

The strength tests performed with the use of the finite element method provided essential information about the strain distribution in the considered body structure. They showed an adequate degree of transducer stress relief, and no cross-sensitivity occurred between various channels. The obtained results form the basis for the proper selection of appropriate points for the positioning of the strain gauge sensors. Moreover, the test results made it possible to evaluate the concurrent effect of a higher number of loads on the body structure. The tests also took into account an extreme example, when all the maximum value loads act on the system. It was found that the structure had adequate strength and that the permissible strain values were not exceeded.

## 5. The Methodology of Processing Information Recorded by the Transducer Sensors

The dependence of the deformation of material used for the construction of the transducer, which showed linear characteristics in relation to the excitation forces, was adopted as the basis for transducer operation. Therefore, the use of resistance strain gauges as sensors was possible. In order to increase the measurement sensitivity and avoid the need to employ a compensation system, it was decided that the four-leg Wheatstone bridge structure would be used in the developed system. To measure the three components, a redundant system in the form of four strain gauge n > 3 bridges was used.

The force vector for the considered identification system of three components takes a general form of $F=\left(F_{y},\;F_{z},\;M_{x}\right)$; therefore, to measure it, at least three strain signals must be recorded $\sigma_{\mathrm{tens}}=\left(\sigma_{1,\;}\sigma_{2},\sigma_{3}\right)$, which were additionally extended by a redundant signal in the adopted solution $\sigma_{4}$.

For conditions defined as described above, the equation system can be expressed in the matrix form (1):

(1) $$\sigma_{tens}\left(t\right)=F\left(t\right)\cdot M$$

where:

$\sigma_{tens}\left(t\right)$—vector of recorded strains,
$F\left(t\right)$—vector of sought for excitations.

The mathematical model of the transducer, which ties the sought-for calculations with the recorded strains, can be presented in the form of the following equation system (2):

(2) $$\left\{\begin{array}{c} \sigma_{1}\left(t\right)=a_{1}\cdot F_{y}\left(t\right)+b_{1}\cdot F_{z}\left(t\right)+c_{1}\cdot M_{x}\left(t\right)\\ \sigma_{2}\left(t\right)=a_{2}\cdot F_{y}\left(t\right)+b_{2}\cdot F_{z}\left(t\right)+c_{2}\cdot M_{x}\left(t\right)\\ \sigma_{3}\left(t\right)=a_{3}\cdot F_{y}\left(t\right)+b_{3}\cdot F_{z}\left(t\right)+c_{3}\cdot M_{x}\left(t\right)\\ \sigma_{4}\left(t\right)=a_{4}\cdot F_{y}\left(t\right)+b_{4}\cdot F_{z}\left(t\right)+c_{4}\cdot M_{x}\left(t\right) \end{array}\right.$$

where:

$\sigma_{1}\left(t\right),\ldots ,\sigma_{3}\left(t\right)$—recorded strains,
$F_{y}\left(t\right),\ldots ,M_{x}\left(t\right)$—excitations acting on the transducer,
$a_{1},\ldots ,c_{4}$–sensitivity coefficients tying strains with loads.

The considered equation system does not have an unambiguous solution because it is an overdetermined system. However, a pseudo-solution can be found for the system, which in accordance with the principles of the adjustment calculus is determined based on the minimum mean square error (MMSE) estimator [24]. Then, equation (3):

(3) $$F^{\prime }\left(t\right)=\left({\left(M^{T}\cdot M\right)}^{-1}\right)\cdot \left(M^{T}\cdot \sigma_{tens}\left(t\right)\right),$$

is a solution indicating property (4):

(4) $${\left(M\cdot F\left(t\right)-\sigma_{tens}\left(t\right)\right)}^{T}-\left(M\cdot F\left(t\right)-\sigma_{tens}\left(t\right)\right)=min.$$

But the solution has a physical sense if the matrix $\left(M_{d}{}^{T}\cdot M_{d}\right)$ is non-singular (5), i.e.,

(5) $$det\left(M^{T}\cdot M\right)\neq 0$$

Equation (5) can be interpreted as follows: the forces operating in the system cause various effects in the form of loads with different values. Otherwise, it would be impossible to determine which load caused the recorded strain increase.

**Figure 10:**
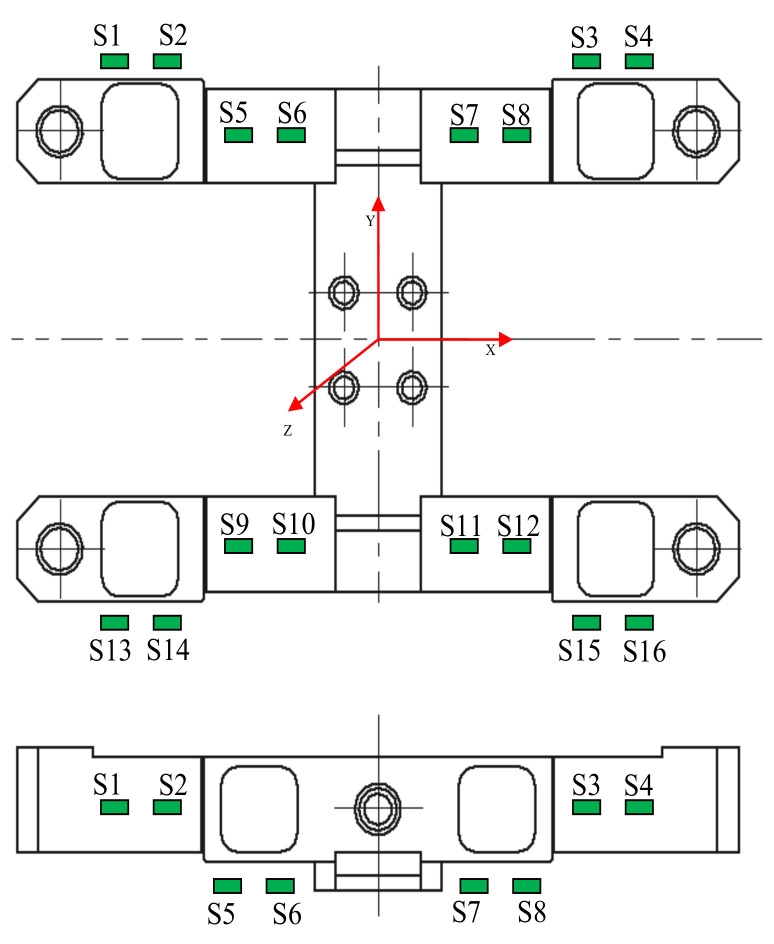
Distribution of strain gauges, where S1, S2, … S16 represent individual strain gauges.

Strain gauge distribution on the transducer construction was shown in Figure 10. In order to measure the $F_{z}$ component, a group of strain gauges numbered S5, S6, S9, S10 was used, whereas, to identify force $F_{y}$, the S1, S2, S3, S4, as well as S13, S14, S15, S16 dcomponent. Strain gauges connected into properly configured Wheatstone bridges made it possible to determine the calibration matrix:

(6) $$\left[\begin{array}{c} M_{1j}\\ M_{2j}\\ M_{3j}\\ M_{4j} \end{array}\right]=\left[\begin{array}{c} \frac{1}{4}{\left(\varepsilon_{6}-\varepsilon_{5}-\varepsilon_{9}+\varepsilon_{10}\right)}_{j}\\ \frac{1}{4}{\left(\varepsilon_{2}-\varepsilon_{1}-\varepsilon_{3}+\varepsilon_{4}\right)}_{j}\\ \frac{1}{4}{\left(\varepsilon_{14}-\varepsilon_{13}-\varepsilon_{15}+\varepsilon_{16}\right)}_{j}\\ \frac{1}{4}{\left(\varepsilon_{7}-\varepsilon_{8}-\varepsilon_{11}+\varepsilon_{12}\right)}_{j} \end{array}\;j=1,\ldots ,4\right]$$

where using $\varepsilon_{n}$, the value of deformation of a single strain gauge was determined.

## 6. Transducer Calibration

It is impossible for theoretical considerations to take into account all the components that affect the final shape of the transducer structure. The inaccuracies related to the manufacturing technology, both of the geometry itself and of the employed structural material or measuring system in the form of strain gauges, the method of adhering, positioning or connecting them, require the final calibration process to be performed before the torque meter is approved for use.

**Figure 11:**
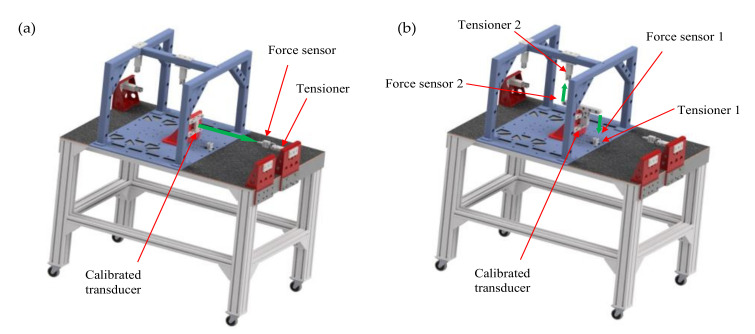
Transducer calibration, (**a**) Fx, (**b**) Mx.

Therefore, the newly-developed construction was subjected to a complete process of identification of the calibration matrix. The process was performed by single value loading of an object with force and torque of predetermined value in the chosen direction, so that other components were eliminated. To this end, a specially designed test stand in the form of a durable table with an extended frame comprising a number of longitudinal members and cross members supported on stable columns was used (Figure 11). The tabletop had numerous fixation holes enabling free and stable positioning of the calibrated transducer. For the performance of excitations, tension screws with integrated single axis force sensors were used.

**Figure 12:**
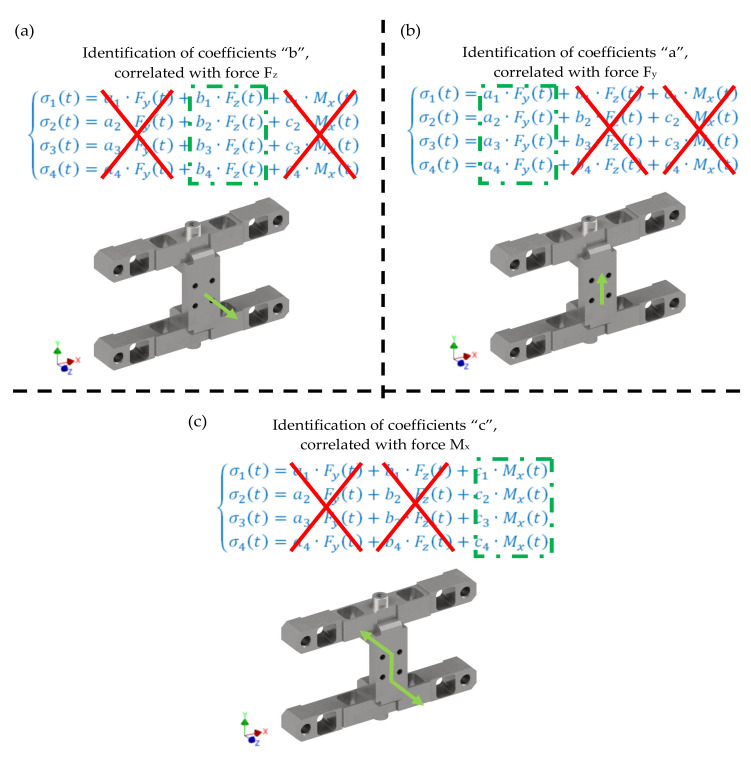
Transducer calibration concept.

The calibration concept of the developed transducer construction was presented in Figure 12. The calibration procedure involves three stages, two of which pertain to the identification of force coefficients along the main default Y, Z axes and a single stage pertaining to the identification of coefficients related to the torque about the X axis.

## 7. Empirical Verification of Mathematical Models

**Figure 13:**
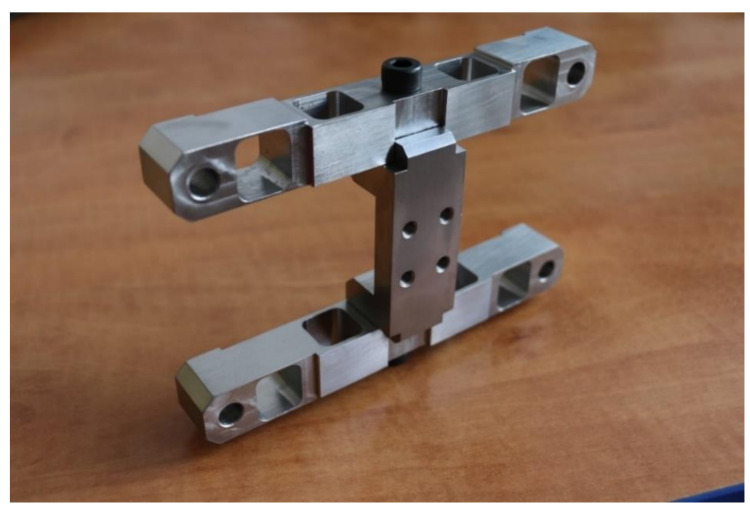
Actual force transducer model.

For the purpose of verification tests, an actual force transducer model was constructed, as shown in Figure 13. It was made as a screwed structure, onto which resistance force transducers were fixed in accordance with the distribution shown in Figure 10.

**Figure 14:**
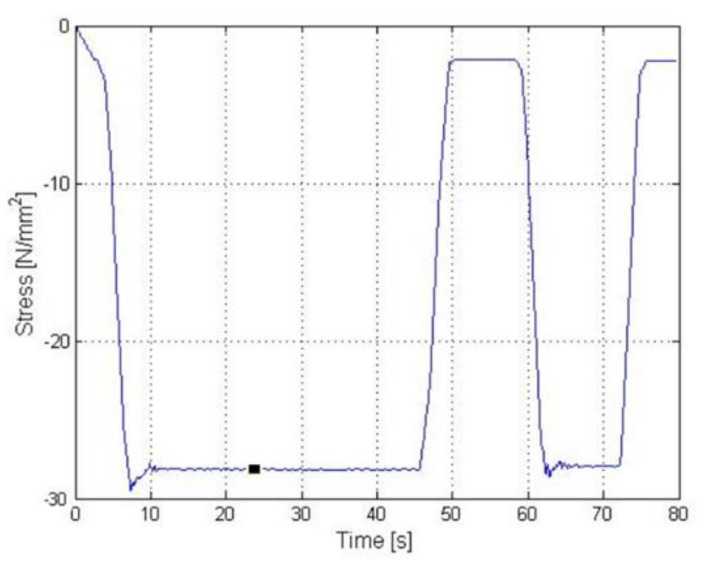
Strain gauge stress measurement result during the Z axis experiment.

**Figure 15:**
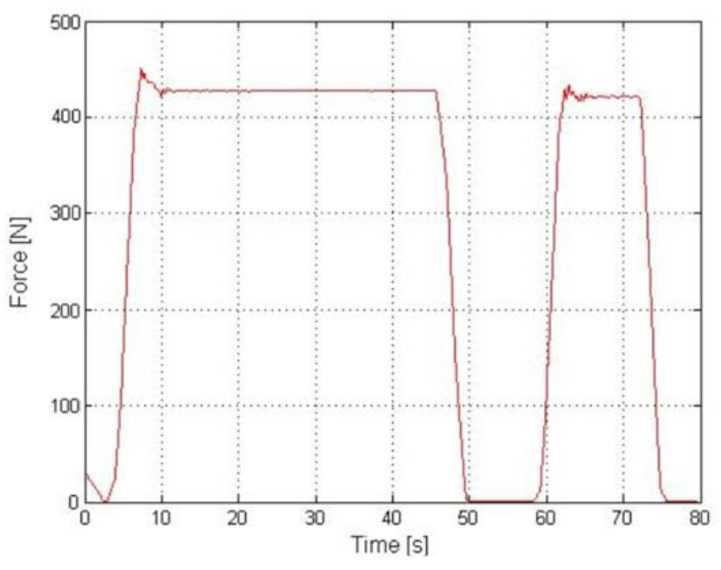
Strain gauge force measurement result during the Z axis experiment.

Example results of the experiment performed for the Z axis on a physical model of a transducer were illustrated in the graphs below. Figure 14 presents the course of strains recorded for one of four strain gauge bridges adhered to the support structure of the transducer. For the purpose of the tests, single-axis strain gauges by Tenmex designated as TFs-5/120 were used. The minimum strain value after the stabilisation of natural vibrations was −2 MPa. For the force measurement, a single axis KT1503 sensor by Megatron (Megatron Elektronik GmbH & Co. KG, Munich, Germany) with a range of 0–5000 N was used. The results of this measurement are presented in Figure 15, where the maximum force was 430 N. By calculating the quotient of these two values, it was possible to determine the coefficient related to the selected bridge for forces acting along the Z axis. When Equation (1) is used, it takes the following value:

(7) $$b_{1}=\frac{-2}{430}=-47\cdot 10^{-4}$$

The procedure was used for the remaining components in accordance with the pattern described above, which made it possible to complete the transducer calibration process.

## 8. Conclusions

The article presents the construction and the method of calibration of a prototype strain gauge transducer for measuring forces acting on the tools of machines operating in the soil. The methodology for processing information collected by strain gauge sensors of the transducer was provided, along with a division into load components. A virtual model of a prototype of such a transducer was also presented and the issues related to shaping its design features were discussed. Moreover, the design of a calibration table was presented.

The basis for the proposed transducer construction is a bar-type body structure fitted with strain gauge sensors. During the design process of the transducer, it was assumed that the system operates in the range of loads causing strains lower than the yield strength. This means that the strains for complex load states are a superposition of strains generated by elementary loads. Such a solution made it possible to give the device body a compact structure.

As mentioned above, the proposed transducer has some limitations; it can be used at low loads ranging from 10 to 600 N, for symmetrically distributed elements working in the clod, such as seeder coulters.

The design process was performed based on simulation methods. The finite element method was used, which enabled effective selection of adequate cross-sections for the employed construction elements. This contributed to the reduction of the construction weight. The strain distributions obtained during the simulation experiments enabled a proper selection of points for fixing the strain gauge transducers.

Particular attention was paid to the essential issue of the precision of transducer indications. The calibration method thoroughly discussed in the article ensures objective assignment of appropriate values to a specific physical action on the structure. The calibration process involved the use of a special test stand, which made it possible to load an object with a force or torque with a set value in the selected direction, while, at the same time, eliminating other components.

The proposed transducer structure was manufactured as a prototype. At the next stage of work, verification work was planned, aimed at a practical evaluation of the parameters selected in the design process of the transducer, in the context of ensuring a linear characteristic of the output signal within the entire measurement range. Furthermore, the precision of the device indications will be determined.

## Data Availability

The data presented in this study are available on request from the corresponding author.
